# Application of different quantitative analytical techniques for estimation of aspirin and omeprazole in pharmaceutical preparation

**DOI:** 10.1186/s13065-022-00854-6

**Published:** 2022-08-15

**Authors:** Ahmed H. Abdelazim, Sherif Ramzy

**Affiliations:** Pharmaceutical Analytical Chemistry Department, Faculty of Pharmacy, Al-Azhar University, Nasr City, Cairo, 11751 Egypt

**Keywords:** Aspirin, Omeprazole, First derivative, TLC densitometry

## Abstract

Several quantitative analytical methods were used to estimate aspirin and omeprazole in recently FDA-approved tablets. The first derivative of the ratio spectra was used to resolve the recorded overlapping spectra between aspirin and omeprazole. The first derivative of the ratio spectra of the studied drug mixtures was divided by a spectrum of a standard solution of omeprazole for the estimation of aspirin. Also, the first derivative of the ratio spectra of the studied drug mixtures was divided by a spectrum of the standard solution of aspirin for the estimation of omeprazole. For the simultaneous quantitative analysis of aspirin and omeprazole, the TLC densitometry technique was applied using TLC aluminum silica gel plates, toluene–acetonitrile–methanol (7:2:0.5, by volume) as the mobile phase, and UV detection at 272 nm. The advantages and disadvantages of the proposed techniques were discussed in the context of the results and the sensitivity limits of the methods. The proposed techniques were validated and successfully applied to the analysis of drugs in pure and pharmaceutical forms. A statistical comparison of the data obtained by the described methods with other data obtained by a previously published HPLC method was performed. The results agreed well with respect to the recommended statistical tests. Furthermore, the greenness of the described methods was assessed using different tools, the analytical eco-scale, the green analytical procedure index and the AGREE evaluation method. The proposed methods showed more adherence to the greenness characters in comparison to the previously reported HPLC method.

## Introduction

Before developing a method for quantitative analysis of raw materials or formulations, many factors must be considered before a particular method is determined and used for a specific recommended application. The first step is to gather information about the compound itself. Accordingly, the type of detection should be selected. UV detection is the most common tool in the field of pharmaceutical analysis.

Recent advances in UV spectroscopic detection of drugs and its application in quantitative analysis have improved the sensitivity of the analytical methods used. UV detection can be performed classically by UV spectrophotometry. It can also be achieved by coupling with HPLC, TLC and capillary electrophoresis techniques, with a significant difference in sensitivity [1–8].

**Fig. 1:**
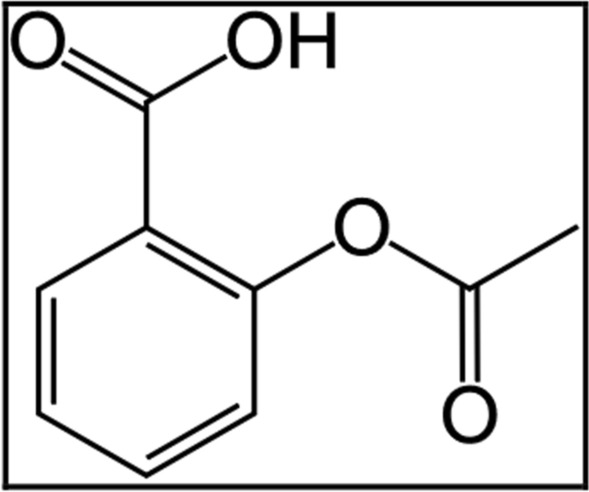
Structural formula of ASP

**Fig. 2:**
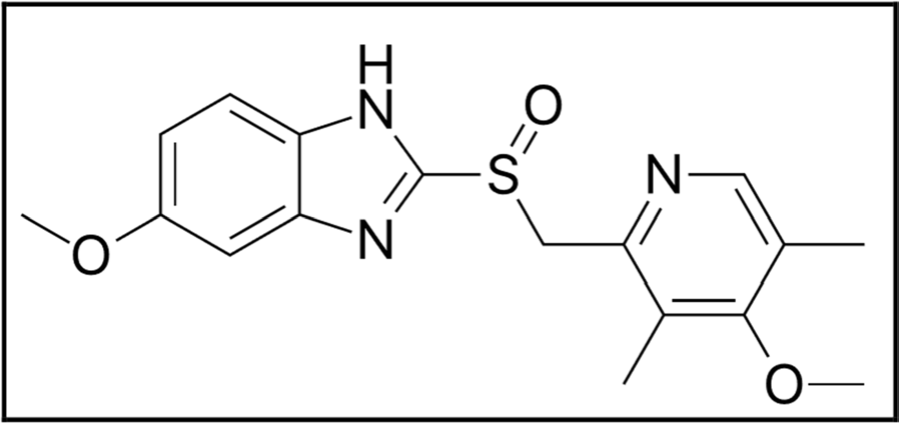
Structural formula of OMZ

Aspirin (ASP), Fig. 1, and omeprazole (OMZ), Fig. 2, are commonly known drugs prescribed for the treatment of common diseases. They have recently been formulated into new pharmaceutical tablets approved by the FDA. The goal of this combination is to reduce the risk of stroke or heart attack in patients who were at risk for developing a peptic ulcer while taking aspirin [9].

In general, an electroanalytical voltammetry technique based on the design of a specific electrode has been described for the determination of ASP and OMZ [10]. On the other hand, high performance thin layer chromatography (HPTLC) [11] and high performance liquid chromatography (HPLC) [12–15] have been published for the determination of the mentioned drugs, but they require sophisticated equipment and long analysis times. Two spectrofluorometric approaches have been recommended for the estimation of ASP and OMZ, but they require the use of specific reagents [16]. Furthermore, spectrophotometric methods based on mathematics, such as the absorption ratio, the multicomponent method, and simultaneous equations, have been used to quantify ASP and OMZ in the [17, 18].

In this work we develop a comparative study for UV detection of the studied drugs using different techniques, classical spectrophotometry and TLC densitometry. TLC densitometry is a simple, straightforward technique for determination of ASP and OMZ. Simultaneous quantitative analysis of ASP and OMZ was developed using TLC aluminum silica gel plates, toluene–acetonitrile–methanol (7:2:0.5, by volume) as a mobile phase, and UV detection at 272 nm. The major advantage of the TLC densitometry method is that it can be performed with a small amount of solvent, which shortens the analysis time and enables cost-effective analytical procedures. The described TLC densitometry was efficiently used for the estimation of the studied drugs at the nanoscale. On the other hand, the first derivative of ratio (^1^DD) spectra method was introduced as a practical, cost-effective analytical procedure to resolve the existing overlap between ASP and OMZ. ^1^DD of the drug mixtures was divided by a spectrum of a standard solution of OMZ for the estimation of aspirin and by a spectrum of the standard solution of ASP for the estimation of OMZ. The applied procedure recommended the use of a derived mathematical tool with specific critical recommendations. Greenness evaluation of the described methods was done using different tools, the analytical eco-scale [19], the green analytical procedure index [20] and the AGREE evaluation method [21]. The proposed methods showed superiority and adherence to the greenness characters in comparison to the previously reported HPLC method.

## Experimental

### Materials and solvents

The pure raw materials ASP (99.23%) and OMZ (99.25%) were kindly provided by Benchmark Health Company, Cairo, Egypt. Yosprala ® tablets, nominally containing 81 mg ASP and 40 mg OMZ per tablet, were kindly provided by Benchmark Health Company, Cairo, Egypt. Methanol, HPLC grade, Sigma-Aldrich, Darmstadt, Germany.

### Instrumentation

UV spectra were measured using Shimadzu UV–Visible 1800 Spectrophotometer (Tokyo, Japan). TLC bands were scanned using CAMAG TLC scanner 3 linked with Linomat 5 auto-sampler (Switzerland). The sample solutions were spotted by autosampler on precoated TLC plates, silica gel 60 GF_254_ 20 × 20 cm (Fluka chemie, Switzerland).

### Standard solutions

A standard stock solution (100 μg/mL) of ASP and OMZ was prepared separately by transferring 10 mg of each drug powder into a 100-mL volumetric flask. The powders were dissolved by adding 50 mL of methanol to each flask and shaking vigorously. The volume was made up to 100 mL with methanol.

### Procedures

#### *First derivative of the ratio spectra (*^*1*^*DD) method*

Two serial dilutions of ASP and OMZ were prepared separately by transferring aliquots corresponding to (200–1400 µg) and (40–200 µg) of ASP and OMZ, respectively, from their standard solutions (100 μg/mL) into two sets of 10-mL volumetric flasks and diluted to volume with methanol. The absorbance spectra of these dilutions were measured against methanol as blank in the wavelength range of 200–400 nm and recorded.

The recorded zero-order absorption spectra of ASP were divided by a suitable absorption spectrum of OMZ to obtain the ratio spectra. Different concentrations of OMZ were tested as divisors. 16 μg/mL OMZ was the optimum in terms of minimal noise of the developed spectral data and highly sensitive results. ^1^DD was manipulated and the amplitudes at 237 nm were proportional to the concentrations of ASP without contribution from OMZ.

On the other hand, the recorded zero-order absorption spectra of OMZ were divided by a suitable absorption spectrum of ASP to obtain the ratio spectra. Different concentrations of ASP were tested as divisors. 40 μg/mL of ASP was the optimum in terms of minimal noise of the developed spectral data and more sensitive results. ^1^DD was manipulated and the amplitudes at 295 nm were proportional to the OMZ concentrations without contribution from ASP.

#### TLC densitometry method

Analysis was performed on pre-coated 20 × 20 cm TLC aluminum silica gel 60 GF254 plates. Samples were applied to the plates using the Linomat 5 auto sampler. The plates were applied 1 cm apart from each other and 1 cm from the bottom edge. The chromatography tank was pre saturated with the mobile phase for 20 min and then developed by ascending chromatography using toluene–acetonitrile–methanol (7:2:0.5, by volume) as the mobile phase.

The plates were dried, detected under UV lamp and scanned under the following conditions:

- Slit dimensions: 6.0 × 0.3 µm
- Wavelength: 272 nm
- Scanning speed: 20 mm/s
- Data resolution: 100 nm/step
- Result output: Integrated band area.

Aliquots of standard solution ASP and OMZ solution (100 μg/mL) corresponding to (10–50 μg) of drug were transferred to a series of 10-mL volumetric flasks and diluted to volume with methanol. 10 L (in triplicate) of each solution was applied to the TLC plate under the specific conditions mentioned. The average band areas of ASP and OMZ were plotted against the corresponding drug concentrations (ng/band) to obtain the calibration curves. The regression parameters were derived. In general, all described procedures were performed in accordance with relevant guidelines.

#### Analysis of laboratory prepared mixtures containing ASP and OMZ

Four synthetic mixtures of ASP and OMZ were prepared. The mixtures were analyzed quantitatively as described under the procedure of each method, and drug concentrations were calculated.

#### Application of the proposed methods to pharmaceutical dosage form

Ten Yosprala® tablets (81 mg ASP and 40 mg OMZ per tablet) were weighed and powdered. The weight of powder corresponding to one tablet was weighed, transferred to a 100 mL volumetric flask, and the volume was made up to 50 mL with methanol. The solution was shaken for 30 min and filtered. The volume was made up to 100 mL with methanol to prepare a stock solution containing 0.81 mg/mL ASP and 0.4 mg/mL OMZ. Five recommended samples of the stock solution were prepared and quantitatively analyzed by the proposed methods.

## Results and discussion

The main purpose of the analyst of pharmaceutical compounds is to develop a technique with higher sensitivity for the detection of the compounds of interest. UV detection is the most commonly used approach in the field of pharmaceutical analysis. Detection or quantification of pharmaceutical compounds can be performed by UV spectrophotometry or coupling with TLC technique, which has a significant impact on sensitivity. In this work, a comparative study for UV detection of the ASP and OMZ using different techniques, classical spectrophotometry and TLC densitometry was developed.

### Description and evaluation of the proposed methods

#### ^*1*^*DD method*

**Fig. 3:**
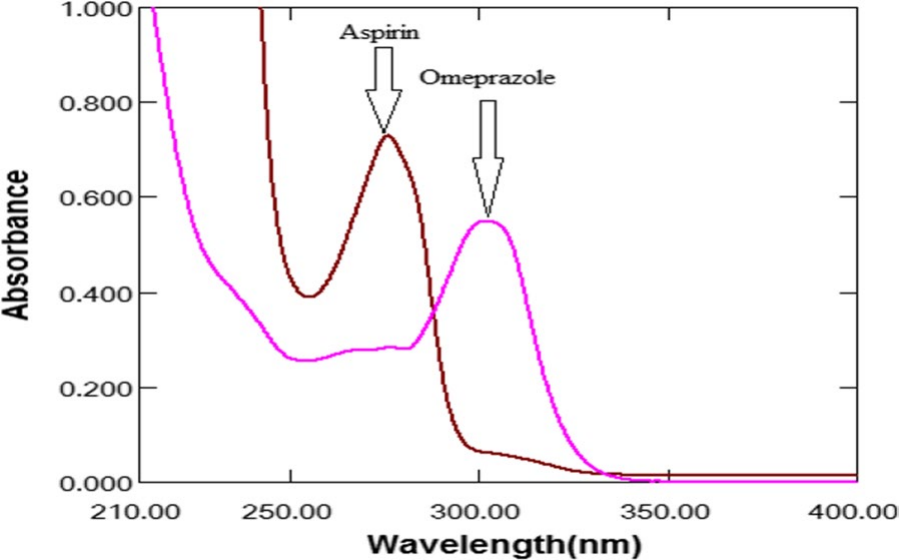
Zero-order absorption spectra of ASP (100 μg/mL) OMZ (10 μg/mL)

The UV absorption spectra of ASP and OMZ (Fig. 3) strongly overlap, so that a direct spectrophotometric analysis is not possible. Theoretically, dividing the absorption spectrum of a compound by a spectrum of the same compound yields a straight line with constant amplitude (parallel to the baseline). However, dividing the absorption spectrum of one compound (X) by the absorption spectrum of another compound (Y) yields a new spectrum, the ratio spectrum. The amplitude of the first or second derivative of the ratio spectrum at a maximum or a minimum is proportional to the concentration of X without interference from Y.

**Fig. 4:**
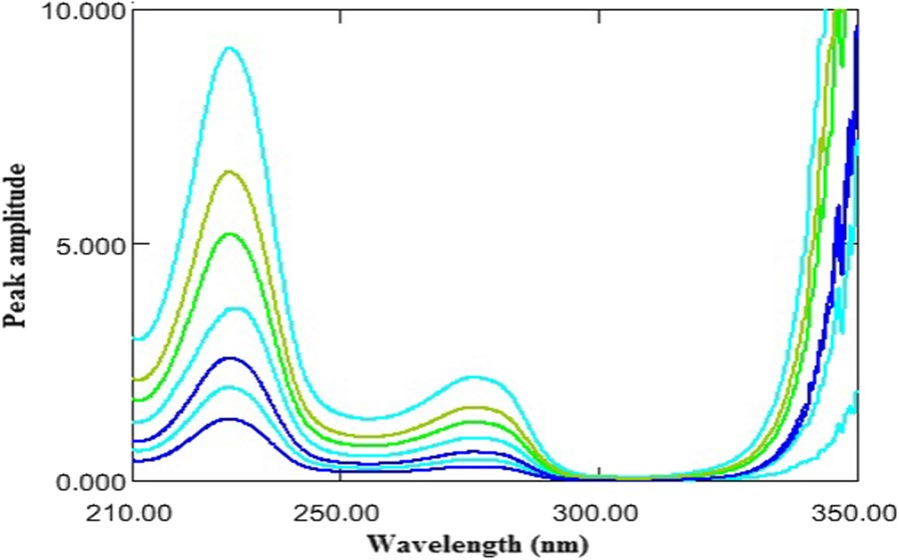
Ratio spectra of ASP (20–140 µg/mL) using 16 µg/mL OMZ as a divisor

By applying the mathematical manipulation of the derivative theory, the absorption spectra of ASP were divided by a suitable absorption spectrum of OMZ to get the ratio spectra (Fig. 4). Selection of the suitable divisor leads to modified signal to noise ratio and high sensitivity output. After testing different divisor concentrations, the optimum results were obtained using 16 μg/mL of OMZ concentration as a divisor.

**Fig. 5:**
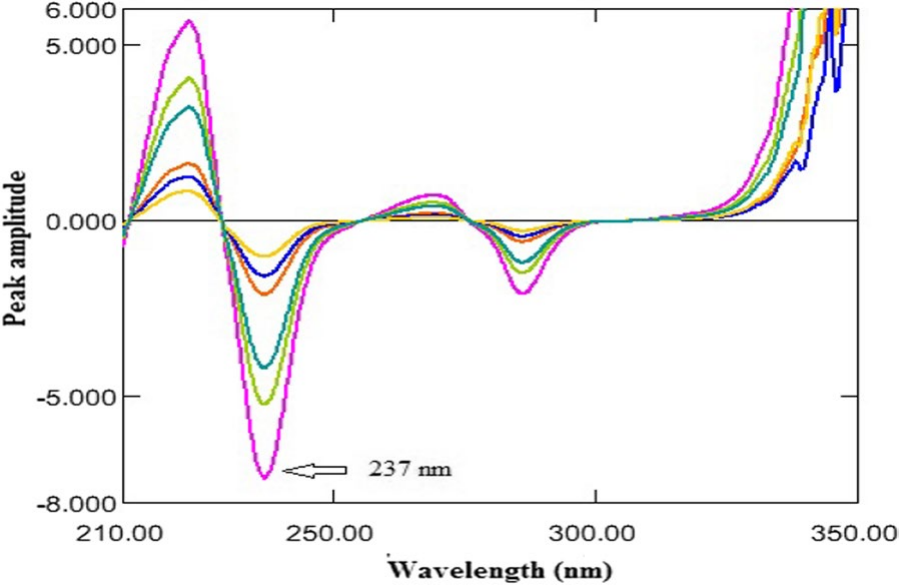
^1^DD of ASP (20–140 µg/mL) using 16 µg/mL OMZ as a divisor

The obtained ratio spectra of ASP were derived to the first orders using Δλ = 2 and scaling factor = 10. The amplitude values of the ratio derivative spectra at 237 nm were proportional to the concentrations of ASP without contribution from OMZ, as shown in Fig. 5.

**Fig. 6:**
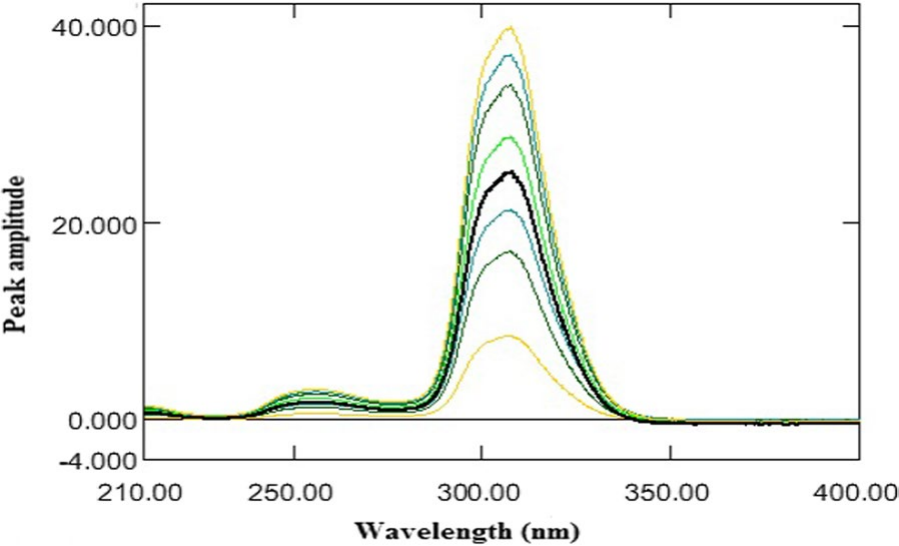
Ratio spectra of OMZ (4–20 µg/mL) using 40 µg/mL ASP as a divisor

**Fig. 7:**
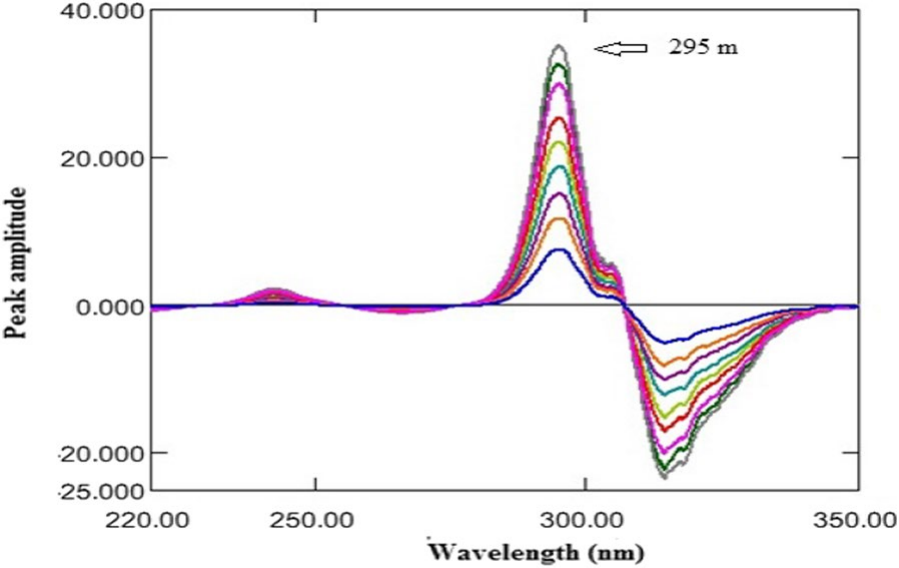
^1^DD of OMZ (4–20 µg/mL) using 40 µg/mL ASP as a divisor

On the other hand, the absorption spectra of OMZ were divided by a suitable absorption spectrum of ASP to get the ratio spectra (Fig. 6). After testing different divisors, 40 μg/mL of ASP was the best regarding to minimum noise of the developed spectral data and highly sensitive results. ^1^DD was manipulated and the amplitudes at 295 nm were proportional to the OMZ concentrations without contribution from ASP, as shown in Fig. 7.

#### TLC densitometry method

**Fig. 8:**
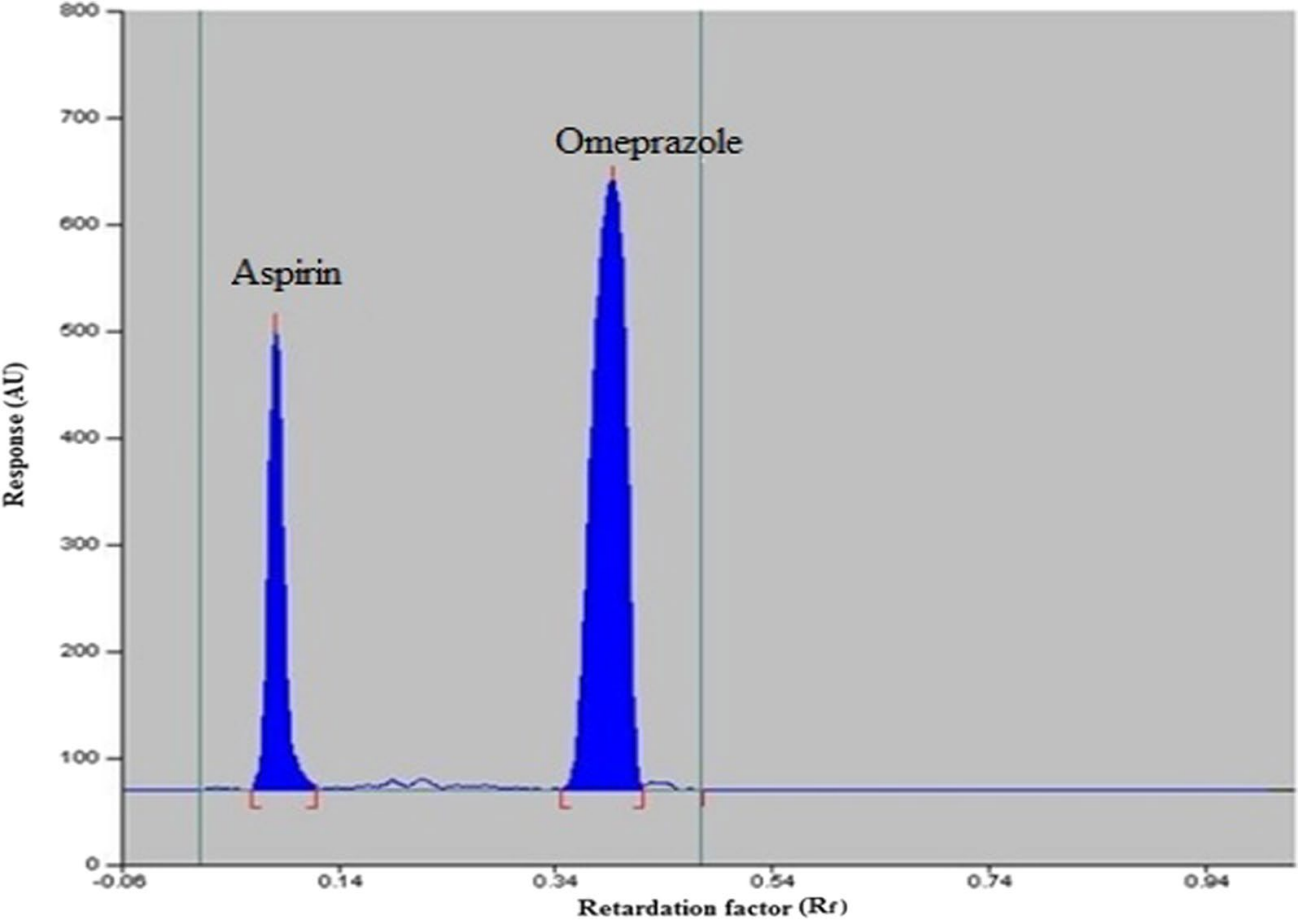
TLC densitogram of ASP (15 ng/band) and OMZ (15 ng/band)

ASP and OMZ solutions were spotted onto the TLC plates and performed in different solvent systems. Solvent systems with different ratios, chloroform–methanol, methanol-toluene, and toluene–acetonitrile, were tested. The mobile phase of toluene: acetonitrile (7: 2, v/v) gave narrow bands of ASP and OMZ with poor resolution and less symmetrical pattern. To improve the resolution of TLC densitometry, a small amount of methanol was added to the mobile phase. The best separation with well-defined bands was obtained after the mobile phase became toluene–acetonitrile–methanol (7:2:0.5, by volume). The chamber was saturated with this mobile phase for 20 min at room temperature. The selected mobile phase allows simultaneous determination of ASP and OMZ without fraying of the separated bands. After optimization of the TLC densitometry conditions, the plates were scanned at 272 nm, where bands appeared at R_f_ of 0.10 for ASP and 0.37 for OMZ as shown in Fig. 8.

To confirm that the TLC densitometry system was working properly during the analysis process, various parameters such as dissolution factor (Rs), retention factor (k‾), and tailing factor (T) were evaluated. The results, presented in Table 1, show that the described TLC densitometry conditions allow complete baseline separation between ASP and OMZ bands with minimal tailing [22].

**Table 1:** System suitability testing parameters of TLC densitometry quantitative analysis of ASP and OMZ

Parameters	Obtained value		Reference value [22]
	ASP	OMZ	
Retardation factor (R_f_)	0.10	0.37	–
Retention factor (K´)	9.00	1.70	1–10
Tailing factor (T)	1.50	1.10	> 2
Resolution (Rs)	4.13		< 2

### Methods validation

The proposed methods were validated according to ICH guidelines [23]. The data of regression equations, limit of detection (LOD), and limit of quantitation (LOQ), accuracy and precision were listed in Table 2. The selectivity of the methods was confirmed by successfully quantitative analysis of ASP and OMZ in their laboratory prepared mixtures (Table 3). The methods were also selectively applied for quantitative determination of ASP and OMZ in the pharmaceutical tablets without interference from tablet matrix, which was assured by the output data of standard addition technique, Table 4.

**Table 2:** Regression and validation data for quantitative analysis of ASP and OMZ by the proposed methods

Parameters	^1^DD		TLC densitometry	
	ASP	OMZ	ASP	OMZ
Wavelength (nm)	237	295	272	272
Linearity range	20–140 µg/mL	4–20 µg/mL	10–50 ng/band	10–50 ng/band
Slope	0.0591	0.2693	478.27	974.66
Intercept	0.0392	0.0780	386.52	133.29
Coefficient of determination (r^2^)	0.9997	0.9999	0.9996	0.9999
LOD	6.183 µg/mL	1.129 µg/mL	3.026 ng/band	2.838 ng/band
LOQ	18.736 µg/mL	3.422 µg/mL	9.169 ng/band	8.597 ng/band
Accuracy (%R)^a^	98.72	100.24	100.38	98.75
Repeatability precision (RSD)^b^	0.621	0.521	0.732	0.528
Intermediate precision (RSD)^b^	0.425	0.670	0.798	0.882

^a^Average of 9 determinations (3 concentrations repeated 3 times)

^b^RSD of 9 determinations (3 concentrations repeated 3 times)

**Table 3:** Quantitative analysis of ASP and OMZ in synthetic laboratory mixtures by the proposed method

^1^DD				TLC densitometry			
Added (µg/mL)		%Recovery		Added (ng/mL)		%Recovery	
ASP	OMZ	ASP	OMZ	ASP	OMZ	ASP	OMZ
20	10	98.25	98.70	20	10	99.35	98.30
24	12	99.96	98.25	24	12	99.08	98.92
32	16	99.31	99.13	32	16	98.91	98.50
40	20	100.22	99.40	40	20	98.03	99.35
Mean ± RSD		99.44 ± 0.879	98.87 ± 0.503	Mean ± RSD		98.84 ± 0.574	98.76 ± 0.466

**Table 4:** Quantitative analysis of ASP and OMZ in Yosprala® tablets by the proposed methods and statistical comparison with the reported method [18]

	%Recovery ± RSD					
	^1^DD		TLC densitometry		Reported method^a^	
	ASP	OMZ	ASP	OMZ	ASP	OMZ
Yosprala ® tablets^b^	100.15 ± 0.621	99.62 ± 0.705	98.27 ± 0.523	99.18 ± 0.689	99.34 ± 0.945	100.01 ± 1.017
Standard addition^c^	98.77 ± 0.621	99.07 ± 0.821	99.25 ± 0.408	98.25 ± 0.258		
*t*-test (2.306)^d^	0.256	0.385	0.327	0.415		
*F*-test (6.388)^d^	1.294	1.186	1.257	1.824		

^a^HPLC determination using C18 column and mobile phase consists of acetonitrile: water; pH 2.9 (60:40, *by volume*) with a flow rate of 1 mL/min and UV detection at 240 nm

^b^Average of five determinations

^c^Average of three determinations

^d^The values in parenthesis are tabulated values of “t” and “F” at (P = 0.05)

### Pharmaceutical applications and statistical comparison

The proposed methods were applied for the quantitative analysis of ASP and OMZ in the pharmaceutical tablets. Statistical comparison of the data obtained by the described methods to another data obtained by previous published HPLC method [18] was achieved. The results were in good agreement, indicating good accuracy and precision of the described methods for the quantitative analysis of the studied drugs in the pharmaceutical dosage form, as shown in Table 4_**.**_

### Comparison and greenness assessment of the proposed methods and reported HPLC method

The present results demonstrated the superiority of the developed TLC method compared to previously published analytical methods in terms of sensitivity, detection limits and quantification. The TLC method allowed the determination of ASP and OMZ over a concentration range of 10–50 ng/band. The lower detection limit of up to 3.026 ng/band for ASP and 2.838 ng/band for OMZ confirmed that TLC method was more sensitive than previously published methods. On the other hand, the ^1^DD spectrophotometric method provided a practical, cost-effective analytical procedure for the determination of ASP and OMZ. The analysis time of the developed methods was shorter and the solvent consumption was much lower than previous works. To compare and determine the greenness ranking of analytical technique, the analytical eco-scale score [19] was calculated based on the amounts of solvent consumed and provides information on the environmental impact of the methods. The achieved score of 78 for TLC and 75 for ^1^DD indicates an excellent green analytical method with minimal negative impact on the environment and human health. The green analytical methods index provided a detailed overview of the different steps of the analytical procedure such as sample preparation, sample handling (collection, preservation, transport and storage), chemicals used and instrumentation. Each factor of the analytical procedures was colored from green to yellow to red to indicate low, medium, and high negative environmental impacts, respectively [20]. The proposed methods had the greenest zones and the fewest red zones (5 green zones and one red zone). In contrast, the reported HPLC method appeared to be less green (4 green zones and 3 red zones). The details obtained from green analytical procedure index and the ease of detection of non-environmentally friendly practices showed that the methods described were superior to the HPLC method. However, the creation of the diagram is time-consuming and complex. Finally, AGREE tool was used [21], which provided the environmental friendliness profile as a numerical value (0.81) for the TLC method, (0.80) for the ^1^DD method, and (0.72) for the described HPLC method, confirming the superiority of the applied method in terms of environmental friendliness. AGREE method combines the advantages and remedies the disadvantages of the aforementioned tools. It takes into account the quantities of reagents, is easy to construct and shows the weaknesses of the studied method. In summary, the results of the assessment tools provided a detailed environmental friendliness profile, complemented each other, and confirmed compliance with environmentally friendly practices in most cases [24, 25]. Comparison and greenness assessment data of the proposed methods and reported HPLC method was presented in Table 5.

**Table 5:** Comparison and greenness assessment of the proposed methods and reported HPLC method

Parameter	TLC densitometry	^1^DD	Reported HPLC [18]
National environmental method index			
Green analytical procedure index			
The AGREE evaluation method			

## Conclusion

In this work, comparative study of the first derivative of the ratio spectra and TLC densitometry methods for the quantitative analysis of ASP and OMZ was developed and discussed. The methods succeeded to quantify the studied drugs in the new combination pharmaceutical dosage form. In addition, greenness ranking assessment of the described methods was done using different tools, the analytical eco-scale, the green analytical procedure index and the AGREE evaluation method. The proposed methods showed more adherence to the greenness characters in comparison to the previously reported HPLC method.

## Data Availability

The datasets used and/or analyzed during the current study are available from the corresponding author on reasonable request.

## Abbreviations

ASP: Aspirin
OMZ: Omeprazole
^1^DD: First derivative of the ratio spectra
